# Extracorporeal Membrane Oxygenation in Children With Cancer or Hematopoietic Cell Transplantation: Single-Center Experience in 20 Consecutive Patients

**DOI:** 10.3389/fonc.2021.664928

**Published:** 2021-04-27

**Authors:** Jenny C. Potratz, Sarah Guddorf, Martina Ahlmann, Maria Tekaat, Claudia Rossig, Heymut Omran, Katja Masjosthusmann, Andreas H. Groll

**Affiliations:** ^1^ Department of General Pediatrics, University Children’s Hospital Münster, Münster, Germany; ^2^ Department of Pediatric Hematology and Oncology, University Children’s Hospital Münster, Münster, Germany

**Keywords:** extracorporeal membrane oxygenation, leukemia, cancer, transplantation, immunosuppression, children, respiratory failure, infection

## Abstract

Extracorporeal membrane oxygenation (ECMO) is a rescue therapy for severe respiratory and/or circulatory failure. Few data exist on the potential benefit of ECMO in immunocompromised pediatric patients with cancer and/or hematopoietic cell transplantation (HCT). Over a period of 12 years, eleven (1.9%) of 572 patients with new diagnosis of leukemia/lymphoma and nine (3.5%) of 257 patients post allogeneic HCT underwent ECMO at our center. Five (45%) and two (22%) patients, respectively, survived to hospital discharge with a median event-free survival of 4.2 years. Experiences and outcomes in this cohort may aid clinicians and families when considering ECMO for individual patients.

## Introduction

Extracorporeal membrane oxygenation (ECMO) technology can provide temporary life support for children with severe respiratory and/or cardiac failure (1). With growing expertise and survival rates of between 40 and 60% overall (1, 2), ECMO has been expanded to children with relevant non-respiratory and non-cardiac co-morbidities (3, 4). Despite an increased risk of life-threatening infections or bleeding due to granulocytopenia and low platelet count, most centers now offer ECMO to children with cancer, and large registries report in-hospital survival rates of 30 to 40% (2–4). In contrast, given the often prolonged and severe immunodeficiency after allogeneic hematopoietic cell transplantation (HCT) with reported in-hospital survival rates of 0 to 20% (4–6), HCT frequently is considered a relative contraindication. Data-driven decision-making to offer or withhold ECMO in patients with cancer or HCT remains difficult, because both groups are extraordinarily heterogeneous and factors predictive of each patients’ relative risk or benefit are currently lacking. Recently, detailed oncological characteristics such as interval from diagnosis, remission status, granulocytopenia and platelet count at ECMO initiation were reported from two large ECMO centers in Europe for nine (7) and twelve (8) patients with hematologic malignancies. Such data may be useful for evaluating patients in the context of decision making for ECMO.

The main objectives of this study were to describe the utilization and outcome of ECMO in children with cancer or HCT at a large pediatric cancer center, and to provide further analyses on indications, co-morbidities, immunodeficiencies and complications in this special population for use in daily practice and future clinical research.

## Patients and Methods

This single center, retrospective cohort study included all patients (<18 years) who were newly diagnosed with a cancer or had received allogeneic HCT at the Department of Pediatric Hematology and Oncology of the University Children’s Hospital Münster between January 2007 (the start of the pediatric intensive care ECMO program) and December 2018. The center’s referral patterns and admission data have been reported recently (9). Patients who had received ECMO were identified through Medical Controlling. ECMO indications followed institutional standards that are based on international guidelines by the Extracorporeal Life Support Organization (available at www.elso.org). Indications were a potentially reversible cause of respiratory and/or circulatory failure, with persistent inadequate gas exchange (such as oxygenation index of >30-40, respiratory acidosis with pH <7.1, harmful ventilator settings, imminent right ventricular failure secondary to pulmonary pressures) and/or high need of vasoactive inotrope medication, together resulting in a mortality risk estimated at ≥80% by an interdisciplinary team of pediatric intensivists and oncologists that assessed ECMO indications and contraindications on a case-by-case basis. All patient-related data was captured by a standardized case report form and entered in pseudonymized form into an electronic database. The study was reviewed and approved by the joint ethics committee of the Westfälische Wilhelms-University of Münster and the Chamber of Physicians Westfalen-Lippe (document 2019-225-f-S).

## Results

During the 12-years study period, 11 of 572 patients with a new diagnosis of leukemia/lymphoma (1.9%; leukemia, 8; lymphoma, 3) and nine of 257 patients post allogeneic HCT (3.5%; MDS/leukemia, 6; non-malignant disorders, 3) underwent ECMO at our center. No single case was identified among patients receiving treatment for solid tumors or brain tumors and among non-transplanted patients with non-malignant hematological disorders. Demographics, key clinical characteristics and outcome of the 20 patients (median age: 11.2 years; r, 0.2-17.8) are summarized in **Table 1**.

**Table 1:** Clinical characteristics of 20 consecutive ECMO patients.

Pt. No.	Year	Age (years)gender (m/f)	Underlying disease	Treatment/protocol prior to ECMO (days post HCT)	Time since diagnosis/start of last treatment (days)	Relevant co-morbidities	Steroid^a^ treatment of disease or co-morbidity	Granulocytopenia at start of ECMO	Type of ECMO support	Indication for ECMO	Steroid^b^ treatment of ECMO indication	Duration of ECMO (days)	ECMO-related complications	Outcome and cause of death
1	2008	8.4 m	ALL (CR1)	Protocol II, ALL-BFM 2000	206/39	None	No	No	R, VV	Pneumonia (Influenza B)	No	21	Infection (sepsis of unknown etiology)	Dead (ECMO withdrawal: secondary sepsis)
2	2010	10.7 f	FA, MDS	HCT (d+483), n/a	983/492	GvHD, BOOP	Yes	No	R, VV	Pneumonia (Influenza A)	No	1	Hemorrhage (cerebral)	Dead (ECMO withdrawal: cerebral hemorrhage)
3	2013	0.6 f	AML M6 (CR1)	ADxE induction, AML-BFM 2012	27/25	None	No	Yes	R, VA	Pneumonia (RSV A)	Yes	9	–	Alive; follow-up: 90 months
4	2014	2.1 f	AML M7 (CR1)	AIE induction, ML-DS 2006	30/29	Trisomy 21	No	Yes	R, VA	Pneumonia (RSV B)	Yes	11	–	Alive; follow-up: 74 months
5	2014	17.7 m	ALL (CR1)	HCT (d+65), ALL SZT-BFM 2003	267/73	AKI	No	No	R, VV	Pneumonia (CMV)	Yes	21	Infection (VRE sepsis), RRT	Dead (ECMO withdrawal: MOF, hypoxic cardiac failure)
6	2014	16.2 m	HD	OEPA, GPOH HD registry	68/19	None	Yes	No	R, VV	Sepsis (*E. coli*), ARDS, pulmonary hemorrhage	No	13	Hemorrhage (pulmonary), RRT	Dead (ECMO withdrawal: MOF, pulmonary failure)
7	2014	1.1 f	AML M2 (CR2)	HCT (d+4), AML SCT-BFM 2007	259/12	None	No	Yes	R, VA	Pneumonia (PIV-3)	Yes	11	Hemorrhage (mucosal, cannula), RRT	Dead (ECMO withdrawal: MOF, abdom. compartment)
8	2015	17.4 f	CHH	HCT (d+32), n/a	253/40	CLD	No	No	R, VV	IPS	Yes	24	Hemorrhage (mucosal), RRT	Dead (ECMO withdrawal: pulmonary fibrosis with PH)
9	2015	15.6 m	ALL (CR1)	HR3, AIEOP ALL-BFM 2009	169/20	None	Yes	Yes	R, VV	Pneumonia (PIV-3), pulmonary hemorrhage	No	15	Infection (*Aspergillus niger*), RRT	Dead (septic shock 3 days after ECMO)
10	2015	11.9 f	ALL (CR1)	Protocol IIIb, AIEOP ALL-BFM 2009	353/9	None	No	Yes	R, VV	Pneumonia (unknown etiology)	No	17	–	Alive; follow-up: 53 months
11	2016	15.3 f	ALL (CR2)	HCT (d+15), ALL SCT 2012 FORUM	582/21	None	No	Yes	R, VV	Pneumonia (HMPV; *Aspergillus fumigatus*)	Yes	34	Infection (*Enterobacter cloacae* sepsis)	Dead (ECMO withdrawal: MOF, pulmonary failure)
12	2016	14.6 m	ALL (CR2)	HCT (d+21), ALL SZT-BFM 2003	904/28	Hepatitis C	No	Yes	R, VV	PERDS	Yes	4	–	Alive; follow-up: 51 months
13	2016	11.6 f	ALL (diagnosis)	n/a	0/n/a	None	No	Yes	R, VV	Pneumonia (*S. aureus*), pulmonary hemorrhage, sepsis	No	48	Infection (SMA), RRT, Hemorrhage (surgery)	Dead (ECMO withdrawal: MOF, pulmonary abscesses)
14	2016	17.8 m	ALL (CR1)	HCT (d+237), ALL SZT-BFM 2003	420/239	GvHD	Yes	Yes	R, VV	Sepsis (3MRGN *E. coli*, SMA), ARDS	No	2	Hemorrhage (pulmonary), RRT	Dead (ECMO withdrawal: pulmonary hemorrhage)
15	2017	15.8 m	LBL (diagnosis)	n/a	0/n/a	Mediastinal mass	No	No	R + C, VA	Mediastinal compression syndrome	No	2	Neurologic (embolic cerebral infarctions)	Alive; follow-up: 33 months
16	2018	0.2 f	Familial HLH	HCT (+19), n/a	93/29	None	No	No	R, VA	IPS	Yes	10	–	Alive; follow-up: 26 months
17	2018	1.7 m	AML M5 (diagnosis)	Prephase, AML-BFM registry	1/1	Leukostasis	No	No	R + C, VA	Pulmonary leukostasis	No	1	Neurologic (infarction)	Dead (ECMO withdrawal: leukostasis)
18	2018	9.8 m	ALL (CR2)	SCA1, IntReALL SR 2010	1350/37	None	Yes	No	R, VV	PERDS, Pneumonia (*Aspergillus fumigatus*)	Yes	27	Infection (CMV), hemorrhage (intestinal)	Dead (on ECMO: MOF, secondary HLH)
19	2018	3.2 m	LBL (CR1)	Protocol II/a, NHL-BFM registry	209/29	None	Yes	No	R + C, VA	Sepsis (Coag. neg. *Staph*), ARDS (Coronavirus OC43)	No	6	–	Alive; follow-up: 23 months
20	2018	5.9 m	SCD	HCT (d+41), n/a	893/48	None	No	No	R, VV	Pneumonia (Bocavirus), diffuse alveolar hemorrhage	Yes	48	–	Dead (ECMO withdrawal: persistent pulmonary failure)

ECMO, extracorporeal membrane oxygenation; HCT, hematopoietic stem cell transplantation; n/a, not applicable; underlying disease: ALL, acute lymphoblastic leukemia; FA, Fanconi anemia; MDS, myelodysplastic syndrome; AML, acute myeloblastic leukemia; HD, Hodgkin disease; CHH, cartilage-hair hypoplasia; LBL, lymphoblastic lymphoma; HLH, hemophagocytic lymphohistiocytosis; SCD, sickle cell disease; CR, complete remission; co-morbidities: GvHD, graft-versus-host-disease; BOOP, bronchiolitis obliterans organizing pneumonia; AKI, acute kidney injury; CLD, chronic lung disease; ECMO support: R, respiratory; C, circulatory; VV, veno-venous cannulation; VA, veno-arterial cannulation; ECMO indication: ARDS, acute respiratory distress syndrome; IPS, idiopathic pneumonia syndrome; PERDS, peri-engraftment respiratory failure; pathogens: RSV, respiratory syncytial virus; CMV, cytomegalovirus; PIV-3, Parainfluenza virus 3; HMPV, human metapneumovirus; MRGN, multidrug-resistant Gram-negative; SMA, Stenotrophomonas maltophilia; Staph, Staphylococcus; VRE, vancomycin-resistant Enterococcus; complications: RRT, renal replacement therapy; cause of death: MOF, multi-organ failure; PH, pulmonary arterial hypertension.

a Treatment with glucocorticosteroids within two weeks prior to ECMO.

b Glucocorticosteroids for treatment of acute illness leading to ECMO.

The median time from the start of the last treatment (chemotherapy or conditioning prior to HCT) to ECMO was 28.2 days (r, -1-492). Six patients had co-morbidities (Down syndrome, 1; chronic graft-versus-host disease (GvHD), 2; mediastinal mass syndrome, 1; leukostasis, 1; other, 3). Six patients had received glucocorticosteroids within the last two weeks before ECMO, four according to the respective chemotherapy protocol (median prednisolone equivalent 2.4 mg/kg/day, median duration of administration 14 days) and two for treatment of chronic GvHD (prednisolone equivalent 0.5 and 1 mg/kg/day for >3 months). Nine patients were granulocytopenic (absolute neutrophil count < 500 cells/µL) at the start of ECMO. All 20 patients required ECMO for respiratory failure, three of them also for concurrent circulatory failure. Acute respiratory failure was due to pulmonary (10) or non-pulmonary (2) infection in 15 patients, and due to the underlying malignancy (2) or HCT-associated inflammatory conditions (3; peri-engraftment respiratory distress syndrome/idiopathic pneumonia syndrome) in the remaining five patients. Ten patients received glucocorticosteroids to treat inflammation in the context of acute respiratory failure (median prednisolone equivalent 2.8 mg/kg/day for a median of three days with a median taper of 18 days).

**Figure 1:**
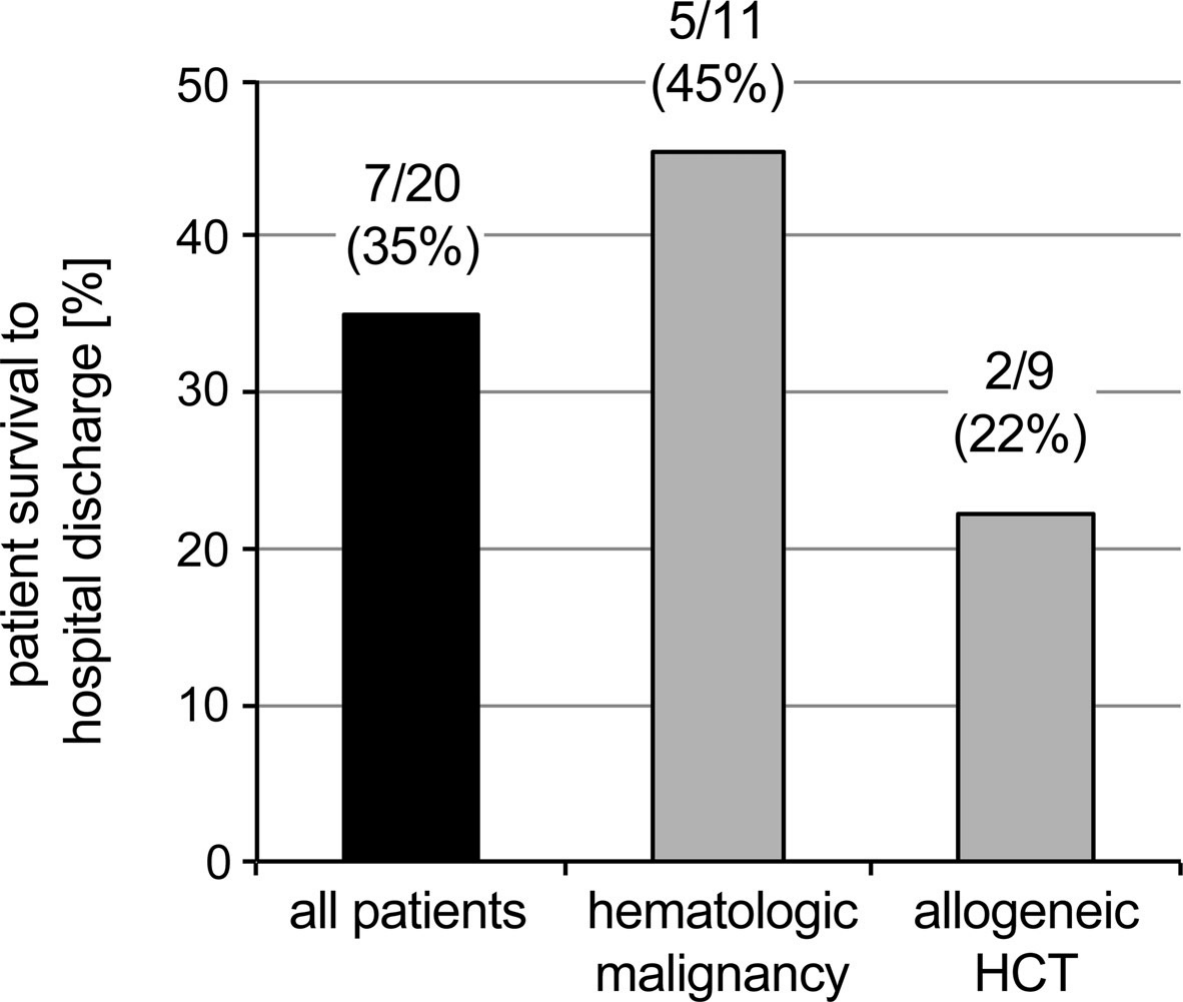
Survival status of ECMO patients. Solid bar: Survival status of the entire cohort. Shaded bars: Survival of patients without allogeneic HCT (left) compared to patients with status post allogeneic HCT (right).

The median duration of ECMO support was 12.2 days (r, 1-48). With the exception of one patient (pt.15, multiple smaller cerebral infarctions, no residual neurologic deficit), complications during ECMO were uniformly associated with death. In two patients, ECMO support was withdrawn within less than 48 hours due to cerebral mass bleeding or leukostasis, respectively. In the remaining 10 non-survivors, ECMO was stopped after a median of 21.4 days (r, 2-48) due to secondary infection (1), pulmonary hemorrhage (1), persistent isolated pulmonary failure (2) and multi-organ failure (6). In the two non-survivors with persistent isolated pulmonary failure, lung damage was considered irreversible on the basis of progressive pulmonary fibrosis and pulmonary hypertension (pt. 8), and diffuse alveolar hemorrhage probably associated with pre-existing sickle cell-associated lung damage underestimated at ECMO indication (pt. 20). One patient died three days after weaning off ECMO from septic shock due to a secondary infection. Seven patients (35%) survived to hospital discharge and are long-term survivors with a median follow-up of 4.2 years (r, 1.9-7.5) (**Figure 1**).

**Supplementary Table 1** shows selected clinical data and scores at baseline and during ECMO tabulated for the entire cohort and according to HCT status. Due to the small number of patients enrolled, no obvious signal for meaningful differences relative to HCT status can be seen. Comparison of survivors and non-survivors, in contrast, shows a trend towards a lower vasoactive inotrope score at ECMO initiation, and shorter duration of granulocytopenia, absence of infectious or bleeding complications, and absence of non-pulmonary organ failures during ECMO in surviving patients (**Supplementary Table 2**).

## Discussion

In this single-center cohort study, the utilization of ECMO in children diagnosed with hematologic malignancies was 1.9%, which is comparable to the 1.3% reported by the Swedish childhood cancer registry (8). With 45% survival to hospital discharge, outcome in our series was similar to the 44% and 50% recently reported from two European high-volume ECMO centers (7, 8). These survival rates are comparable to other high-risk ECMO settings such as for pertussis, cardiopulmonary resuscitation or neonatal cardiac failure (1). A summary of selected studies reporting ECMO outcomes in children with hematologic malignancies or allogeneic HCT is provided in **Supplementary Table 3**. In patients post allogeneic HCT, the use of ECMO at our center was approximately two-fold higher than in patients with hematological malignancies (3.5%). Two of nine patients (22%) survived to discharge, which is within the range of data from the U.S. Pediatric Health Information System database and the Extracorporeal Life Support Organization (ELSO) registry (4, 6, 11). The fact that all seven patients surviving to discharge also are long-term survivors is notable considering the high rates of mortality reported within the first 90 days post ECMO treatment (12, 13).

While, despite signals for improved survival of ECMO in more recent years (10, 11), status post allogeneic HCT, a diagnosis of leukemia, and granulocytopenia remain to be generally associated with higher odds of mortality relative to non-immunocompromised patients (4), clinical variables in our patients at initiation and during ECMO do not show any evidence for additional differences between patients post allogeneic HCT and those receiving chemotherapy for hematological malignancies. Indeed, although not reaching statistical significance, exploration of differences between survivors and non-survivors suggest a low vasoactive inotrope score (VIS) at initiation of ECMO, shorter duration of granulocytopenia, absence of emerging infections and bleeding complications, and absence of non-pulmonary organ failure during ECMO as being associated with survival.

Existing scores that incorporate cancer as global high-risk diagnosis when estimating mortality at ICU admission or ECMO initiation, such as PIM3 (14), P-PREP (15) or Ped-RESCUERS (16), did not distinguish survivors and non-survivors in our cohort. To better predict chances of survival, scores capturing information that is more relevant to the status of patients with hematological malignancies and allogeneic HCT in the context of intensive care support and ECMO would be highly desirable, and well-established Pediatric Oncology databases and study groups may be able to contribute detailed and robust data, specifically on pre-ECMO variables. At present, consistent with our experience, the development of new infections, major hemorrhage, and organ complications while on ECMO seem to be the main variables associated with unfavorable outcome (2, 7, 8, 17), and multi-organ failure is a leading cause of ECMO withdrawal and death (2, 6, 7). In this context, the systematic use of scores [e.g. daily PELOD-2 (18)] could help to identify and compare guidance for stopping of ECMO across centers.

Obvious limitations of the current analysis include its retrospective, single-center format with analysis of a small cohort of inhomogeneous patients that precludes robust statistical analyses of unfavorable outcome; and the absence of detailed data on functional, cognitive, behavioral and quality of life outcomes in the surviving patients. Despite these limitations, the analysis of this cohort of unselected, consecutive patients supports the notion that ECMO can offer a chance for survival to children with hematological malignancies or allogeneic HCT who are treated in curative intention and develop respiratory and/or cardiovascular failure due to a presumably reversible acute disease process. Nevertheless, morbidity and mortality of this invasive rescue therapy remain high. As recently stated (19), to offer or withhold ECMO in immunocompromised children with potentially reversible respiratory or cardiorespiratory failure should therefore remain a careful patient- and family-centered decision made with the support of a multidisciplinary expert team.

## Data Availability Statement

The raw data supporting the conclusions of this article will be made available by the authors, without undue reservation.

## Ethics Statement

The studies involving human participants were reviewed and approved by the ethics committee of the Westfälische Wilhelms-University of Münster and the Chamber of Physicians Westfalen-Lippe (document 2019-225-f-S). Written informed consent from the participants’ legal guardian/next of kin was not required to participate in this study in accordance with the national legislation and the institutional requirements.

## Author Contributions

JP, KM, and AG contributed to conception and design of the study. JP, SG, MA, MT, CR, and HO acquired data. JP, SG, and AG analyzed the data. JP performed the statistical analysis. JP, SG, KM, and AG interpreted the data. JP, KM, and AG supervised the project. JP and AG wrote the manuscript. All authors contributed to manuscript revision, read, and approved the submitted version.

## Conflict of Interest

The authors declare that the research was conducted in the absence of any commercial or financial relationships that could be construed as a potential conflict of interest.
