# Supplementary material for: Extracorporeal Membrane Oxygenation in Children With Cancer or Hematopoietic Cell Transplantation: Single-Center Experience in 20 Consecutive Patients
Source: Front Oncol. 2021 Apr 27;11:664928. doi: 10.3389/fonc.2021.664928 (PMC8111086; doi:10.3389/fonc.2021.664928)
Supplement: Supplementary file 1 [file DataSheet_1.pdf]

Supplementary Material

1 Supplementary Tables

1.1 Supplementary Table 1. Clinical variables of ECMO patients without HCT compared to ECMO patients with prior HCT

Variable	All (n=20)		Patients without HCT (n=11)		Patients with HCT (n=9)		*P-value*[a]
	Median (IQR) or number (%)	n with data	Median (IQR) or number (%)	n with data	Median (IQR) or number (%)	n with data	
Pre ECMO							
Duration of granulocytopenia (days)	23.2 (15.6-40.3)	8	14.4 (11.4-18.6)	4[b]	45.7 (31.8-57.4)	4	***0.058***
Platelet count (x 10^3 /µl)	66 (28-105)	18	72 (34-172)	10	63 (27-86)	8	*0.474*
Days on mechanical ventilation	1.5 (0.3-2.9)	20	0.3 (0.1-2.0)	11	1.8 (1.3-3.1)	9	*0.236*
Days on inhaled nitric oxide	0.8 (0.3-1.7)	10	0.9 (0.3-2.1)	5	0.7 (0.3-1.7)	5	*0.762*
pH	7.25 (7.19-7.33)	19	7.26 (7.10-7.34)	10	7.25 (7.22-7.28)	9	*0.735*
Oxygenation index	40 (33-46)	17	43 (34-46)	10	37 (30-44)	7	*0.617*
PIM3 score[c]	19 (9-24)	20	22 (12-31)	11	18 (8-21)	9	*0.360*
P-PREP score[d]	45 (42-57)	19	48 (43-56)	10	45 (42-57)	9	*0.528*
Ped-RESCUERS[d]	54 (47-62)	17	53 (45-58)	10	58 (53-64)	7	*0.352*
VIS score[e]	9 (2-26)	16	2 (0-28)	9	13 (4-15)	7	*0.854*
During ECMO							
Duration of granulocytopenia (days)	4.1 (2.6-6.2)	7	4.1 (2.9-7.2)	5	2.9 (1.8-4.0)	2[f]	*0.571*
Platelet count (x 10^3 /µl)	43 (34-70)	19	39 (22-64)	10	51 (37-71)	9	*0.412*
No. of platelet transfusion per day	1.0 (0.7-2.1)	20	1.2 (0.9-2.5)	11	0.7 (0.5-1.2)	9	*0.080*
New onset of infection	6 (30%)	20	4 (36%)	11	2 (22%)	9	*0.642*
Major bleeding[g]	7 (37%)	19	3 (30%)	10	4 (44%)	9	*0.650*
Renal replacement therapy	7 (37%)	19	3 (30%)	10	4 (44%)	9	*0.650*
No additional organ failure	9 (45%)	20	5 (45%)	11	4 (44%)	9	*1.000*
One additional organ failure[h]	4 (20%)	20	2 (18%)	11	2 (22%)	9	*1.000*
≥ 2 additional organ failures[h]	7 (33%)	20	4 (36%)	11	3 (33%)	9	*1.000*
ECMO duration (days)	12.2 (5.3-22.0)	20	13.4 (7.2-11.0)	11	10.8 (4.0-23.8)	9	*1.000*

ECMO, extracorporeal membrane oxygenation; HCT, hematopoietic cell transplantation; IQR, interquartile range.

[a] for categorical covariates, univariate differences in survival were calculated using Fisher's exact test. For continuous data, univariate differences in survival were assessed using Wilcoxon's rank sum test.

[b] one additional patient with unknown duration of neutropenia at presentation of acute leukemia.

[c] PIM3, Pediatric Index of Mortality 3, predicting mortality (%) at pediatric intensive care unit admission. (14)

[d] P-PREP, Pediatric Pulmonary Rescue with ECMO Prediction Score,(15) and Ped-RESCUERS, Pediatric Risk Estimation Score of Children Using Extracorporeal Respiratory Support,(16) of in-hospital mortality (%) at ECMO initiation.

[e] VIS, vasoactive inotrope score.(12)

[f] two additional non-survivors with HCT did not reach granulocyte recovery.

[g] according to definitions of the Extracorporeal Life Support Organization registry.

[h] Organ failures on ECMO in 6 patients without HCT were cardiac/circulatory (5), kidney (4), liver (2), ileus/intestinal ischemia (2); and in 5 patients with HCT were cardiac/circulatory (5), kidney (3), liver (1), ileus/intestinal ischemia (2).

1.2 **Supplementary Table 2.** Clinical variables in ECMO survivors compared to ECMO non-survivors

**Variable**	**All (n=20)** Median (IQR) or number (%)	**n with data**	**Survivors (n=7)** Median (IQR) or number (%)	**n with data**	**Non-survivors (n=13)** Median (IQR) or number (%)	**n with data**	***P-value***[a]
Pre ECMO							
Duration of granulocytopenia (days)	23.2 (15.6-40.3)	8	19.8 (15.2-22.9)	4	45.7 (29.1-57.4)	4[b]	*0.200*
Platelet count (x 10^3 /µl)	66 (28-105)	18	72 (38-167)	6	63 (23-105)	12	*0.423*
Days on mechanical ventilation	1.5 (0.3-2.9)	20	0.3 (0.3-4.2)	7	1.6 (0.2-2.9)	13	*0.891*
Days on inhaled nitric oxide	0.8 (0.3-1.7)	10	1.0 (0.3-2.7)	4	0.8 (0.3-1.35	6	*0.658*
pH	7.25 (7.19-7.33)	19	7.24 (7.18-7.28)	7	7.27 (7.21-7.36)	12	*0.635*
Oxygenation index	40 (33-46)	17	42 (36-47)	7	37 (27-46)	10	*0.617*
PIM3 score[c]	19 (9-24)	20	22 (20-24)	7	14 (8-34)	13	*0.473*
P-PREP score[d]	45 (42-57)	19	45 (44-48)	7	50 (42-60)	12	*0.510*
Ped-RESCUERS[d]	54 (47-62)	17	54 (48-57)	7	56 (48-67)	10	*0.402*
VIS score[e]	9 (2-26)	16	0 (0-11)	6	14 (4-35)	10	***0.051***
During ECMO							
Duration of granulocytopenia (days)	4.1 (2.6-6.2)	7	2.6 (1.9-3.2)	4	7.2 (6.2-13.3)	3[f]	***0.057***
Platelet count (x 10^3 /µl)	43 (34-70)	19	37 (34-65)	7	51 (32-69)	12	*0.578*
No. of platelet transfusion per day	1.0 (0.7-2.1)	20	1.2 (0.8-1.8)	7	0.9 (0.7-2.1)	13	*0.699*
New onset of infection	6 (30%)	20	0 (0%)	7	6 (46%)	13	***0.052***
Major bleeding[g]	7 (37%)	19	0 (0%)	7	7 (58%)	12	***0.017***
Renal replacement therapy	7 (37%)	19	0 (0%)	7	7 (58%)	12	***0.017***
No additional organ failure	9 (45%)	20	7 (100%)	7	2 (15%)	13	***<0.001***
One additional organ failure[h]	4 (20%)	20	0 (0%)	7	4 (31%)	13	*0.249*
≥ 2 additional organ failures[h]	7 (33%)	20	0 (0%)	7	7 (54%)	13	***0.045***
ECMO duration (days)	12.2 (5.3-22.0)	20	8.6 (4.9-10.2)	7	20.8 (10.8-27.0)	13	*0.109*

ECMO, extracorporeal membrane oxygenation; HCT, hematopoietic cell transplantation; IQR, interquartile range.
[a] for categorical covariates, univariate differences in survival were calculated using Fisher's exact test. For continuous data, univariate differences in survival were assessed using Wilcoxon's rank sum test.
[b] one additional patient with unknown duration of neutropenia at presentation of acute leukemia.
[c] PIM3, Pediatric Index of Mortality 3, predicting mortality (%) at pediatric intensive care unit admission. (14)
[d] P-PREP, Pediatric Pulmonary Rescue with ECMO Prediction Score,(15) and Ped-RESCUERS, Pediatric Risk Estimation Score of Children Using Extracorporeal Respiratory Support,(16) of in-hospital mortality (%) at ECMO initiation.
[e] VIS, vasoactive inotrope score.(12)
[f] two additional non-survivors did not reach granulocyte recovery.
[g] according to definitions of the Extracorporeal Life Support Organization registry.
[h] Organ failures on ECMO in 11 non-survivors were cardiac/circulatory (10), kidney (7), liver (3), ileus/intestinal ischemia (4).

1.3 **Supplementary Table 3.** Survival to hospital discharge in selected studies reporting ECMO outcome for children with hematologic malignancy and/or HCT

**Study**	**Year**	**Study Type**				**Hematologic malignancy**		**Allogeneic HCT**		**All patients**		**General PICU control**	
		**Re-gistry**	**Cli-nical**	**Multi-center**	**Single-center**	**n**	**survival**	**n**	**survival**	**n**	**survival**	**n**	**survival**
Wolfson et al.[a] °*	2005	✓		✓		18	4 (22 %)	11	1 (9 %)	29	5 (17 %)	--	--
Gow et al.°*	2006	✓		✓		--	--	12	0 (0 %)	--	--	--	--
Gupta et al.[b] °*	2008	✓		✓		49	14 (29 %)	17	0 (0 %)	66	14 (21 %)	2696	1550 (57%)
Gow et al.°*	2009	✓		✓		73	29 (40 %)	--	--	--	--	ND	45-56%
Zabrocki et al.[c] °*	2011	✓		✓		84	ND (30%)	22	1 (5%)	106	ND (25%)	2109	63 %
Di Nardo et al.[d] *	2014	✓		✓		--	--	29	3 (10 %)	--	--	--	--
Cortina et al.[e]	2018		✓		✓	9	4 (44 %)	--	--	--	--	--	--
Coleman et al.[f] °	2020	✓		✓		120	42 (35 %)	31	6 (19 %)	151	48 (32 %)	--	--
Ranta et al.	2020		✓	✓		12	6 (50 %)	--	--	--	--	ND	68 %
Steppan et al.[g]	2020	✓		✓		10	7 (70 %)	8	4 (50 %)	18	11 (61 %)	415	249 (61 %)
Bridges et al.[h] °	2021		✓	✓		17	6 (35 %)	9	3 (33 %)	26	9 (35 %)	174	72 %
Olson et al.[i] °*	2021	✓		✓		--	--	51	6 (12 %)	--	--	--	--

Note: these selected studies are not result of a systematic database search. ND, no data.
° Subgroups of interest were extracted from a broader study population.
* These analyses are based on the same registry (Extracorporeal Life Support Organization).
[a] Study did not differentiate type of malignancy, all are included.
[b] Study included respiratory failure only; "hematologic malignancy" comprises leukemia, lymphoma, aplastic anemia, agranulocytosis, excluding other cancers.
[c] Study included respiratory failure only; type of malignancy not differentiated, all are included.
[d] Transplant type was not specified, based on diagnoses assumed allogeneic.
[e] Study included respiratory failure only.
[f] Study did not differentiate type of HCT, all patients with "bone marrow transplant" are included.
[g] Study did not differentiate outcome by type of malignancy or HCT, 6 patients with solid tumors and 1 autologous and 1 unknown HCT, respectively, are included.
[h] Veno-venous ECMO only; survival is to intensive care unit discharge.
[i] 32 of these patients were treated between 2010-2019, including all 6 survivors.
